# Parametrial involvement in cervical cancer is associated with systemic inflammatory indices: a retrospective observational study

**DOI:** 10.3389/fmed.2026.1842260

**Published:** 2026-06-25

**Authors:** Carlo Ronsini, Antonino Di Nuzzo, Giuseppe Cucinella, Maria Cristina Solazzo, Eva Iannacone, Federica Gherardi, Vito Chiantera

**Affiliations:** 1 Unit of Gynecologic Oncology, National Cancer Institute, IRCCS, Fondazione "G. Pascale", Naples, Italy; 2 Unit of Gynecology and Obstetrics, Policlinico“G. Martino”, Department of Human Pathology of Adult and Childhood “G. Barresi”, University of Messina, Messina, Italy; 3 Radiation Oncology Unit, Istituto Nazionale Tumori Istituto di Ricovero e Cura a Carattere Scientifico Istituto di Ricovero e Cura a Carattere Scientifico (IRCCS) Fondazione G. Pascale, Naples, Italy; 4 Department of Woman, Child and General and Specialized Surgery, University of Campania “Luigi Vanvitelli”, Unit og Gynaecology and Obstetrics, Naples, Italy

**Keywords:** cervical cancer, lymphovascular space invasion (LVSI), parametrial invasion, systemic inflammatory indices, tumor-related inflammation

## Abstract

**Objective:**

The aim of this study is to evaluate the association between parametrial invasion (PI) and systemic inflammatory indices in patients with cervical cancer, exploring whether routinely available hematological markers reflect local tumor spread.

**Methods:**

A retrospective observational study was conducted including patients with cervical cancer treated with surgery and/or radiotherapy and/or chemotherapy between July 2023 and December 2025. Patients were stratified according to the presence or absence of PI, as determined by histopathological or radiological assessment. Systemic inflammatory indices derived from pre-treatment blood tests included neutrophil-to-lymphocyte ratio (NLR), monocyte-to-lymphocyte ratio (MLR), platelet-to-lymphocyte ratio (PLR), systemic inflammatory response index (SIR), and systemic inflammation response index (SIRI). Comparisons between groups were performed using non-parametric tests. Receiver operating characteristic (ROC) curves were used to evaluate the standalone discriminative ability of each inflammatory index. Logistic regression models were applied to assess associations with PI. To reduce information loss and limit model instability in this exploratory cohort, inflammatory indices were analyzed as continuous variables. Adjusted analyses were performed using separate clinically predefined models, each including one inflammatory index together with depth of stromal infiltration and fornix involvement.

**Results:**

A total of 79 patients were included in the analysis. PLR, MLR, NLR, and SIRI were significantly higher in patients with PI compared with those without PI, whereas SIR did not differ significantly. In analyses stratified by the extent of PI, significant differences were observed for MLR, NLR, and SIRI. In unadjusted logistic regression models, PLR, MLR, NLR, and SIRI were associated with PI. After adjustment for depth of stromal infiltration and fornix involvement in separate clinically predefined models, only MLR remained significantly associated with PI (adjusted OR per 0.1-unit increase: 1.986; 95% CI: 1.095–3.603; *p* = 0.024). ROC analysis showed modest standalone discriminative ability, with all AUC slightly below 0.75.

**Conclusion:**

Parametrial invasion in cervical cancer is associated with measurable alterations in systemic inflammatory indices, reflecting the host response to locoregional tumor spread. Among the evaluated indices, MLR retained an association with PI after adjustment for clinically relevant local spread parameters. However, the modest standalone discriminative performance and exploratory nature of the analysis preclude the use of these indices as independent diagnostic tools. Further prospective validation is required before any clinical application.

## 1 Introduction

Parametrial invasion represents a key determinant in cervical cancer, as it directly defines the FIGO staging system stage and consequently guides therapeutic decision-making and prognosis (1). Accurate assessment of parametrial involvement is therefore essential in the diagnostic work-up of affected patients (2). In clinical practice, parametrial invasion is evaluated through a combination of gynecological examination and imaging techniques, including transvaginal ultrasound and advanced modalities such as magnetic resonance imaging (MRI) and positron emission tomography (PET) (3). While these approaches ensure high diagnostic accuracy, they are associated with significant costs and limited availability in low-resource settings. This issue is particularly relevant considering that cervical cancer remains disproportionately prevalent in low- and middle-income countries, with the highest incidence rates reported in sub-Saharan Africa, Central America, and Southeast Asia (4). In these regions, access to advanced diagnostic tools is often restricted due to financial and infrastructural limitations. In this context, the evaluation of cost-effective and easily accessible biomarkers may contribute to a better understanding of the biological and inflammatory correlates of local tumor spread. In recent years, increasing evidence has highlighted the role of systemic inflammatory indices as potential markers of tumor aggressiveness and local invasion in several solid malignancies (5). For instance, elevated neutrophil-to-lymphocyte ratio (NLR) and platelet-to-lymphocyte ratio (PLR) have been associated with poor prognosis and advanced disease in colorectal, stomach, lung, and breast cancers (6–8), while indices such as the systemic inflammatory response index (SIR), and systemic inflammation response index of inflammation (SIRI) have shown correlations with tumor progression and metastatic potential in hepatocellular carcinoma and pancreatic cancer (9). These findings suggest that systemic inflammation may reflect tumor–host interactions involved in cancer dissemination. In cervical cancer, similar mechanisms may underlie the association between inflammatory status and local tumor spread, including parametrial invasion. However, the clinical utility of these indices in this specific setting remains to be fully elucidated. Importantly, the evaluation of systemic inflammatory indices is not intended to replace radiological imaging or to define clinically applicable diagnostic thresholds. Rather, these indices were investigated as low-cost markers potentially reflecting the systemic inflammatory response associated with local tumor spread.

### 1.1 Objectives

The aim of this study is to evaluate the association between parametrial invasion and systemic inflammatory indices, including neutrophil-to-lymphocyte ratio (NLR), monocyte-to-lymphocyte ratio (MLR), platelet-to-lymphocyte ratio (PLR), systemic inflammation response index (SIRI), and the systemic inflammatory response index (SIR), in patients with cervical cancer.

To this end, we conducted a retrospective analysis of patients treated at our center with surgery and/or radiotherapy and/or chemotherapy. Patients were stratified into two groups based on the presence or absence of parametrial invasion, as determined by histopathological assessment when available or by radiological evaluation in patients not undergoing surgery, to investigate whether these inflammatory indices differed significantly between the groups.

## 2 Materials and methods

### 2.1 Study design

A retrospective observational study was conducted to examine the relationship between systemic inflammatory indices and parametrial involvement (PI) in patients with cervical cancer [24]. The primary outcome was the comparison of the distributions of neutrophil-to-lymphocyte ratio (NLR), monocyte-to-lymphocyte ratio (MLR), platelet-to-lymphocyte ratio (PLR), systemic inflammatory response index (SIR), and systemic inflammation response index (SIRI) between patients with and without PI. Secondary exploratory outcomes included the comparison of inflammatory indices according to the extent of PI, the assessment of their association with PI using logistic regression models, and the descriptive evaluation of their unadjusted standalone discriminative ability using receiver operating characteristic (ROC) analysis. PI was used as the grouping variable for the primary comparative analysis and as the dependent variable in the logistic regression models. The study was not designed to derive clinical thresholds or to develop a prediction model for parametrial involvement.

### 2.2 Settings

The study was conducted at IRCCS Istituto Nazionale Tumori “G. Pascale” in Naples and at the University of Campania “Luigi Vanvitelli,” using a prospectively maintained institutional database of patients treated between July 2023 and December 2025. The study protocol received approval from the Ethics Committee of the University of Campania “Luigi Vanvitelli,” with protocol code 0035558/i, on November 24, 2022, and adhered to the Declaration of Helsinki. The study was registered on ClinicalTrials.gov under the identifier NCT05673252. Those data were derived from a retrospective evaluation of additional parameters of the registered protocol.

### 2.3 Study population

Patients were included if they met all of the following criteria: age equal to or greater than 18 years; diagnosis of cervical cancer confirmed either histologically or radiologically; availability of complete clinical, laboratory, and tumor-related data; and availability of blood tests performed within 7 days prior to treatment initiation.

**Figure 1:**
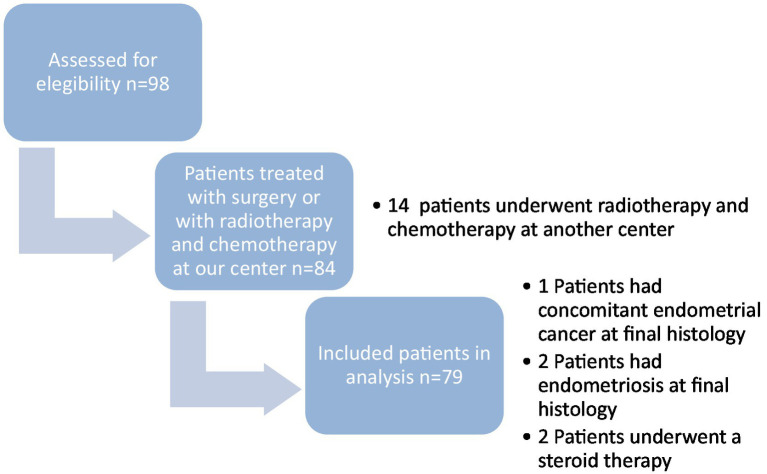
CONSORT flow diagram.

Patients were excluded in the presence of chronic inflammatory conditions, including inflammatory bowel disease (IBD), autoimmune or rheumatological disorders, and endometriosis; in the presence of synchronous malignancies or a history of malignancy within the previous 3 years; in case of conditions affecting endogenous corticosteroid levels; or in case of steroid therapy within 30 days prior to enrollment. Only patients fulfilling all inclusion criteria and none of the exclusion criteria were retained for analysis (Figure 1).

### 2.4 Variables

Parametrial involvement was the principal grouping variable for the primary outcome analysis and was defined as present or absent; for secondary exploratory analyses, PI was further categorized as monolateral or bilateral. PI was established nbased of histopathological findings, when available. In patients not undergoing surgery, radiological assessment by magnetic resonance imaging (MRI) and/or positron emission tomography (PET) was used. Clinical examination alone was not considered sufficient unless supported by imaging. Inflammatory indices were used as exposure variables and derived from routine blood tests. These included the neutrophil-to-lymphocyte ratio (NLR), calculated as the ratio of neutrophils to lymphocytes; the monocyte-to-lymphocyte ratio (MLR), calculated as the ratio of monocytes to lymphocytes; the platelet-to-lymphocyte ratio (PLR), calculated as the ratio of platelets to lymphocytes; the systemic inflammatory response index (SIR), defined as neutrophils multiplied by platelets divided by lymphocytes; and the systemic inflammation response index (SIRI), calculated as neutrophils times monocytes divided by lymphocytes. All inflammatory indices were derived from blood samples collected before treatment initiation, ensuring that systemic inflammatory markers were not influenced by subsequent therapeutic interventions. Additional variables were collected to describe the study population and adjust multivariate models, including age, body mass index (BMI), menopausal status, prior abdominal surgery, comorbidities, treatment type, stromal infiltration, fornix involvement, lymphovascular space invasion (LVSI) (10), lymph node status, histotype, and grade. Among these variables, only depth of stromal infiltration and fornix involvement were included as clinically predefined covariates in the revised adjusted models, because they represent anatomical features of local tumor spread most directly related to parametrial involvement. PI and lymph node status were assessed through radiology when histopathological confirmation was unavailable, with evaluations independently performed by two radiologists blinded to laboratory data. In patients not undergoing surgery, radiological assessment (MRI and/or PET) was used. In the present cohort, PI was assessed by histopathology in 77 patients (97.5%) and by radiological evaluation in 2 patients (2.5%). Clinical examination alone was not considered sufficient unless supported by imaging. Histopathological diagnoses were confirmed by two pathologists blinded to clinical and laboratory information.

### 2.5 Laboratory procedures

Peripheral blood samples were collected within 7 days before treatment started using standard venipuncture with ethylenediaminetetraacetic acid (EDTA) tubes. All samples were processed according to routine laboratory protocols at each participating center, with consistent timing between collection and analysis. Hematological parameters were measured with automated analyzers and reported as absolute counts in cells per microliter. Internal and external quality control procedures were implemented to ensure measurement consistency and reproducibility across centers.

### 2.6 Statistical analysis

Continuous variables were summarized using medians and interquartile ranges (IQR), while categorical variables were described by counts and percentages. For the primary outcome analysis, the distributions of the five inflammatory indices were compared between patients with and without PI using the Wilcoxon rank-sum test. Comparisons of other continuous and categorical baseline variables employed the Wilcoxon rank-sum test and the Chi-square or Fisher’s exact test, as appropriate. For the secondary exploratory analysis based on the extent of PI, the Kruskal–Wallis test was used. The null hypothesis for the primary outcome stated that there was no difference in the distribution of each inflammatory index between patients with and without parametrial involvement. Statistical significance was defined as a *p*-value less than 0.05, representing a 5% chance of type I error. Throughout the manuscript, *p*-values were reported to three decimal places, except for values below 0.001, which were reported as *p* < 0.001. Univariate linear regression models were initially developed using each inflammatory index as a dependent variable and parametrial involvement as the independent grouping variable, in order to quantify differences in index levels according to PI status. Afterward, ROC curves were generated for each inflammatory index to provide an exploratory description of its unadjusted standalone discriminative ability between patients with and without PI. Area under the curve (AUC) values were interpreted descriptively only. No optimal cut-off points, sensitivity/specificity estimates based on selected thresholds, or threshold-derived classifications were retained in the revised analysis, since the study was not designed to develop or validate a diagnostic prediction tool. Accordingly, all regression analyses used inflammatory indices as continuous variables. Logistic regression models were then fitted using parametrial involvement as the dependent variable and each inflammatory index as a continuous exposure variable. To improve clinical interpretability, odds ratios were reported for predefined increments of each index: 50 units for PLR, 0.1 units for MLR, 1 unit for NLR, 500 units for SIR, and 1 unit for SIRI. Given the limited sample size and the exploratory purpose of the study, five separate adjusted models were developed, each including only one inflammatory index together with depth of stromal infiltration and fornix involvement. These covariates were selected *a priori* because they represent clinically relevant anatomical features of local tumor spread directly related to parametrial involvement. This strategy was adopted to avoid overparameterization, reduce the risk of overfitting, and avoid data-driven variable selection procedures in a relatively small cohort. Results are reported as odds ratios (OR) with 95% confidence intervals (CI) and corresponding *p*-values. All statistical analyses were performed using R software (Version 2024.12.0 + 467). Given the retrospective design and fixed available sample size, no *post-hoc* power analysis was used to support the robustness of the findings. The precision of the estimates was instead interpreted through effect sizes and their 95% confidence intervals, and the adjusted analyses were intentionally restricted to clinically predefined parsimonious models.

### 2.7 Risk of bias

To minimize potential sources of bias while avoiding overfitting, adjusted analyses were restricted to clinically predefined covariates representing local anatomical tumor spread. Specifically, each inflammatory index was assessed in a separate adjusted model including depth of stromal infiltration and fornix involvement. No data-driven covariate selection procedure was applied. Data extraction and analysis were independently conducted by two investigators, one of whom was blinded to the study objectives. The use of predefined inclusion and exclusion criteria, along with a homogeneous cohort, helped reduce confounding factors. No missing data were present for the variables included in the main analyses.

## 3 Results

### 3.1 Characteristics

A total of 79 patients were included in the analysis. Baseline clinical, demographic, and tumor-related characteristics are reported in Table 1.

**Table 1:** Clinical, demographic and tumor-related characteristics according to parametrial involvement.

Characteristic	Bilateral, *N* = 22^1^	Monolateral, *N* = 19^1^	None, *N* = 38^1^	*p*-value^2^
Age	59, (16)	58, (18)	51, (15)	0.015
BMI	25.0, (3.3)	25.0, (4.8)	25.5, (8.0)	0.900
Missing	11	8	2	
Menopouse	18, (82%)	15, (79%)	19, (50%)	0.017
Previous Abdominal Surgery	11, (50%)	5, (26%)	25, (66%)	0.019
Comorbidity	14, (64%)	10, (53%)	12, (32%)	0.054
Missing	0	0	1	
Treatment				<0.001
Chemo-Radiation	19, (86%)	18, (95%)	12, (32%)	
LPT	2, (9.1%)	1, (5.3%)	15, (39%)	
MIS	1, (4.5%)	0, (0%)	11, (29%)	
Histotype				0.007
Adenocarcinoma	0, (0%)	0, (0%)	8, (21%)	
Adeno-Squamous	0, (0%)	2, (11%)	1, (2.6%)	
Squamous	22, (100%)	17, (89%)	29, (76%)	
Grading				0.700
1	1, (4.5%)	1, (5.3%)	5, (13%)	
2	6, (27%)	4, (21%)	11, (29%)	
3	15, (68%)	14, (74%)	22, (58%)	
Stromal Infiltration				0.110
Superficial	3, (14%)	3, (16%)	14, (37%)	
Middle	3, (14%)	4, (21%)	9, (24%)	
Deep	16, (73%)	12, (63%)	15, (39%)	
Positive Fornix	16, (73%)	12, (63%)	6, (16%)	<0.001
LVSI	15, (68%)	11, (58%)	27, (71%)	0.600
Pelvic Lymphnodes	15, (68%)	12, (63%)	15, (39%)	0.060
Aortic Lymphnodes	5, (23%)	2, (11%)	0, (0%)	0.005

1 Median, (IQR); n, (%).

2 Kruskal-Wallis rank sum test; Pearson’s Chi-squared test; Fisher’s exact test [35].

Patients with parametrial involvement (PI) were older compared to those without PI (59 vs. 51; *p* = 0.015) and more often postmenopausal (82% vs. 50%; *p* = 0.017). Previous abdominal surgery was less common in patients with PI (50% vs. 66%; *p* = 0.019), whereas comorbidities showed a trend but did not reach statistical significance (64% vs. 32%; *p* = 0.054). Treatment distribution differed significantly between groups (chemo-radiation 86% vs. 32%; *p* < 0.001). Histotype distribution was also significantly different (squamous 100% vs. 76%; *p* = 0.007), while grading showed no statistically significant differences. Regarding parameters of documented tumor progression, fornix involvement was significantly more frequent in patients with PI (73% vs. 16%; *p* < 0.001), and aortic lymph node positivity was more common in patients with PI (23% vs. 0%; *p* = 0.005). No significant differences were noted for stromal infiltration, LVSI, or pelvic lymph node status.

### 3.2 Outcomes

**Figure 2:**
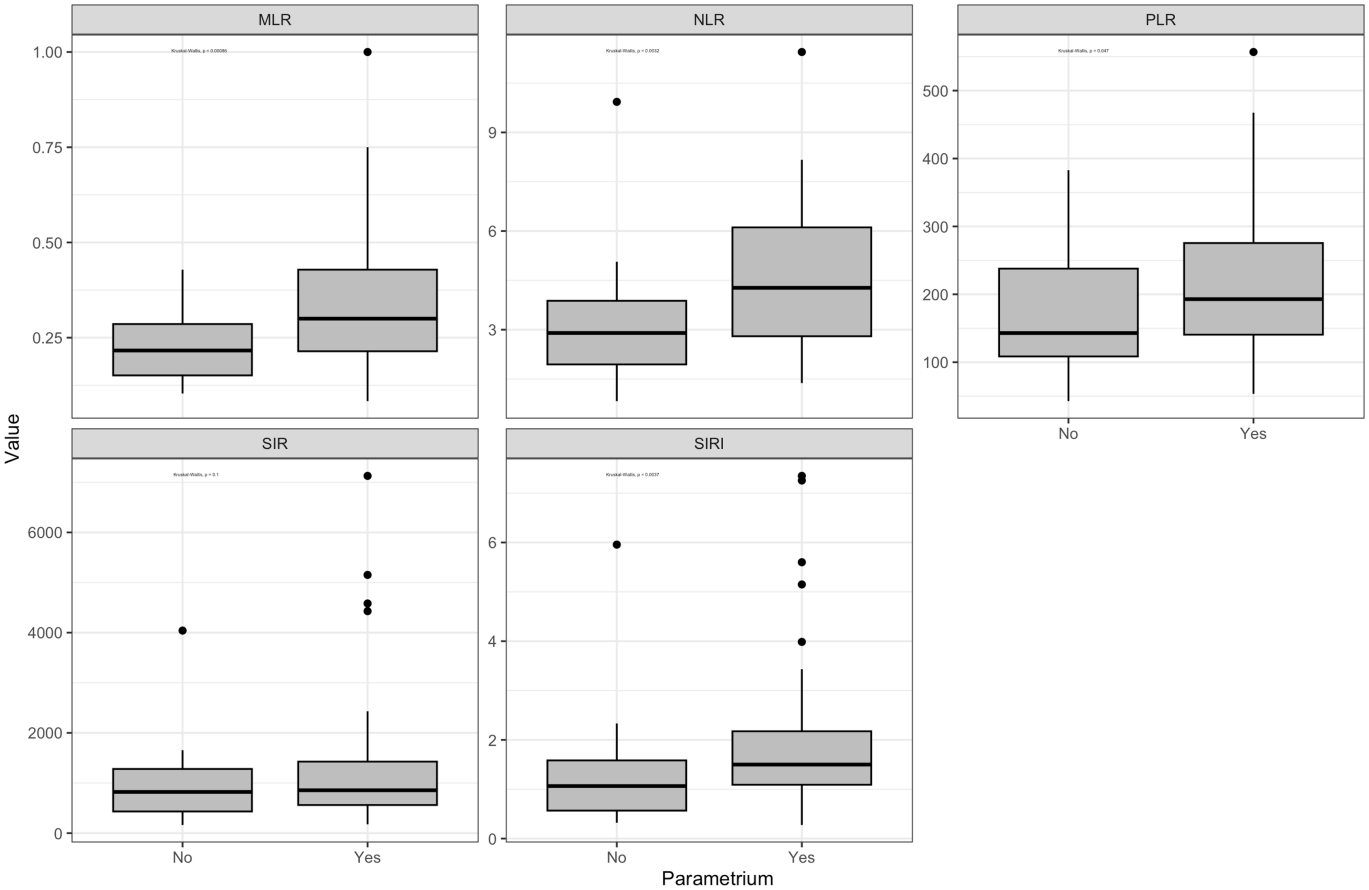
Distribution of inflammatory indices according to parametrial involvement. PLR was higher in patients with PI compared to those without (193 vs. 143; *p* = 0.047). MLR showed a significant increase in patients with PI (0.30 vs. 0.22; *p* < 0.001), as did NLR (4.27 vs. 2.90; *p* = 0.003). SIRI was also significantly higher in patients with PI (1.50 vs. 1.06; *p* = 0.004), whereas SIR did not differ significantly (856 vs. 822; *p* = 0.10).

The primary outcome analysis, consisting of the comparison of inflammatory index distributions between patients with and without parametrial involvement, is reported in Table 2 and visually summarized in Figure 2.

**Table 2:** Distribution of inflammatory indices according to parametrial involvement.

Characteristic	No, *N* = 38^1^	Yes, *N* = 41^1^	*p*-value^2^
PLR	143, (129)	193, (135)	0.047
MLR	0.22, (0.13)	0.30, (0.21)	<0.001
NLR	2.90, (1.93)	4.27, (3.31)	0.003
SIR	822, (849)	856, (866)	0.100
SIRI	1.06, (1.02)	1.50, (1.08)	0.004

1 Median, (IQR).

2 Wilcoxon rank sum test [25].

A subgroup analysis by the extent of PI is reported in Table 2.1.

**Table 2.1:** Distribution of inflammatory indices according to the extent of parametrial involvement.

Characteristic	Bilateral, *N* = 22^1^	Monolateral, *N* = 19^1^	None, *N* = 38^1^	*p*-value^2^
PLR	196, (142)	193, (86)	143, (129)	0.130
MLR	0.30, (0.15)	0.33, (0.22)	0.22, (0.13)	0.004
NLR	3.93, (3.21)	4.42, (2.88)	2.90, (1.93)	0.012
SIR	787, (775)	1,024, (1,184)	822, (849)	0.300
SIRI	1.47, (0.9)	1.72, (1.23)	1.06, (1.02)	0.014

1 Median, (IQR).

2 Kruskal-Wallis rank sum test.

MLR was significantly associated with the extent of parametrial involvement (0.30 vs. 0.33 vs. 0.22; *p* = 0.004), and NLR also showed significant differences across categories (3.93 vs. 4.42 vs. 2.90; *p* = 0.012); Same pattern for SIRI (1.47 vs. 1.72 vs. 1.06; *p* = 0.014). No statistically significant associations were found for PLR and SIR.

### 3.3 Regression models and exploratory ROC analysis

PI was associated with higher PLR, MLR, NLR, and SIRI values in univariate linear regression models, as detailed in Table 3.

**Table 3:** Univariate linear regression models of inflammatory indices according to parametrial involvement.

Characteristic	Beta	95% CI^1^	*p*-value
PLR	48	2.2, 95	0.040
MLR	0.12	0.06, 0.19	<0.001
NLR	1.4	0.54, 2.3	0.002
SIR	497	−18, 1,012	0.058
SIRI	0.81	0.19, 1.43	0.011

1 CI, Confidence Interval.

**Figure 3:**
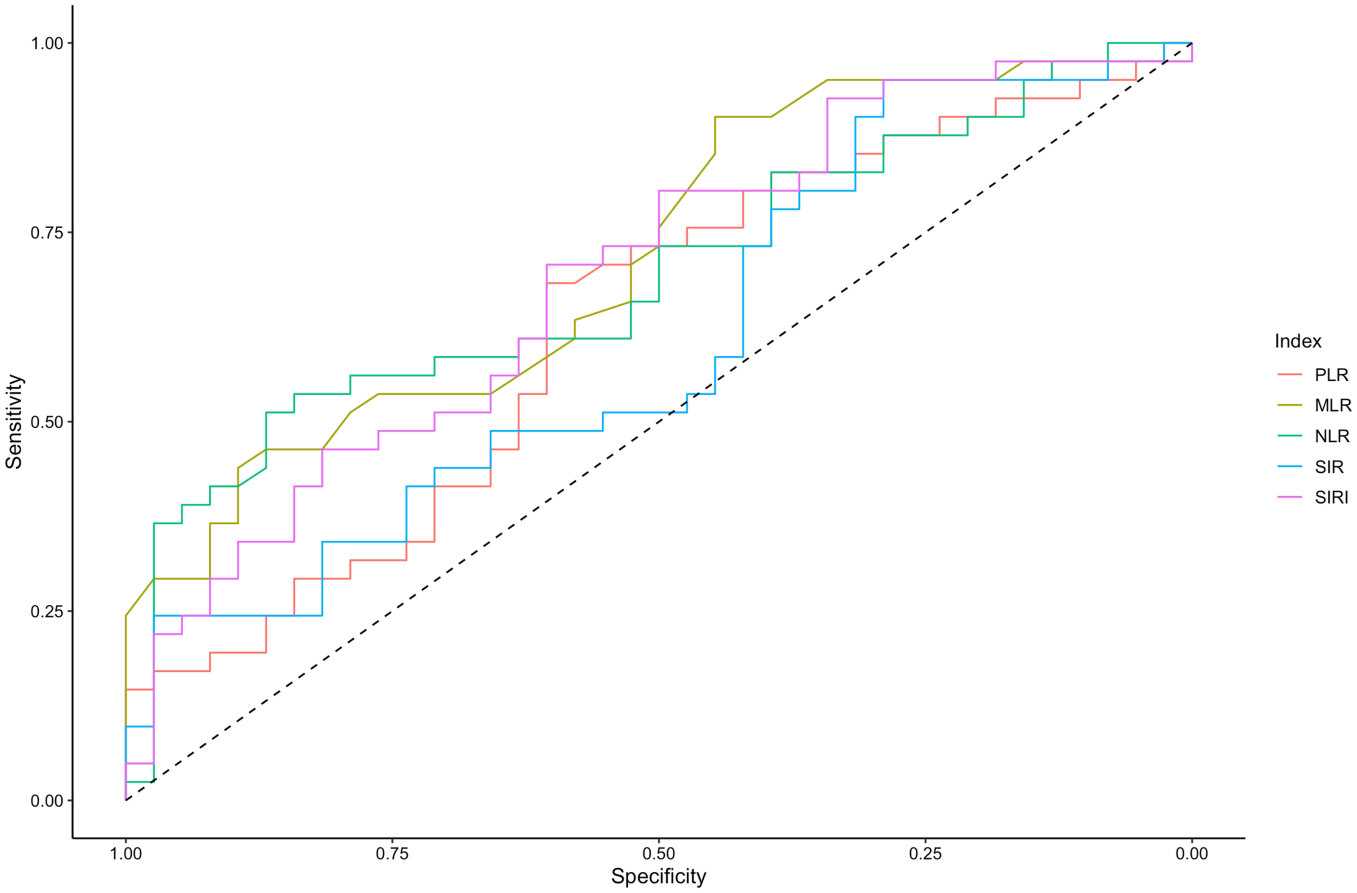
Exploratory receiver operating characteristic curves of inflammatory indices according to parametrial involvement. Exploratory ROC analysis showed modest unadjusted standalone discriminative ability for all inflammatory indices. MLR showed the highest AUC value (AUC = 0.718), followed by NLR (AUC = 0.693), SIRI (AUC = 0.690), PLR (AUC = 0.630), and SIR (AUC = 0.607). No threshold-based diagnostic measures were retained in the revised analysis, since the present study was not designed to derive or validate clinically applicable cut-off values.

PLR was associated with PI (*β* = 48; 95% CI 2.2–95; *p* = 0.040), MLR was linked to PI (*β* = 0.12; 95% CI 0.06–0.19; *p* < 0.001), and NLR was connected to PI (*β* = 1.4; 95% CI 0.54–2.3; *p* = 0.002). SIRI was also related to PI (*β* = 0.81; 95% CI 0.19–1.43; *p* = 0.011), while SIR did not reach statistical significance (*β* = 497; 95% CI − 18–1,012; *p* = 0.058). The unadjusted discrimination of inflammatory indices by PI status was assessed using ROC analysis, as shown in Figure 3.

To avoid information loss and the risk of overfitting associated with data-derived dichotomization, inflammatory indices were analyzed exclusively as continuous variables in logistic regression models. Each index was evaluated in a separate adjusted model including depth of stromal infiltration and fornix involvement as clinically predefined covariates, as shown in Table 4. These models were designed to estimate associations rather than to generate a diagnostic prediction tool.

**Table 4:** Separate logistic regression models evaluating continuous inflammatory indices associated with parametrial involvement.

Inflammatory index	Unit of increase	Unadjusted OR, 95% CI	*p*-value	Adjusted OR, 95% CI	*p*-value
PLR	Per 50-unit increase	1.273 (1.003–1.615)	0.047	1.116 (0.804–1.550)	0.512
MLR	Per 0.1-unit increase	2.117 (1.307–3.430)	0.002	1.986 (1.095–3.603)	0.024
NLR	Per 1-unit increase	1.519 (1.137–2.030)	0.005	1.339 (0.928–1.932)	0.119
SIR	Per 500-unit increase	1.270 (0.968–1.667)	0.085	1.086 (0.784–1.504)	0.619
SIRI	Per 1-unit increase	1.770 (1.070–2.930)	0.026	1.395 (0.838–2.322)	0.201

Each adjusted odds ratio was derived from a separate logistic regression model including one inflammatory index, depth of stromal infiltration, and fornix involvement. OR, odds ratio; CI, confidence interval; PLR, platelet-to-lymphocyte ratio; MLR, monocyte-to-lymphocyte ratio; NLR, neutrophil-to-lymphocyte ratio; SIR, systemic inflammatory response index; SIRI, systemic inflammation response index.

In unadjusted logistic regression models using continuous inflammatory indices, increasing PLR, MLR, NLR, and SIRI values were significantly associated with parametrial involvement. Specifically, the odds of PI increased for each 50-unit increase in PLR (OR 1.273; 95% CI 1.003–1.615; *p* = 0.047), each 0.1-unit increase in MLR (OR 2.117; 95% CI 1.307–3.430; *p* = 0.002), each 1-unit increase in NLR (OR 1.519; 95% CI 1.137–2.030; *p* = 0.005), and each 1-unit increase in SIRI (OR 1.770; 95% CI 1.070–2.930; *p* = 0.026). SIR was not significantly associated with PI in unadjusted analysis. In separate adjusted models including depth of stromal infiltration and fornix involvement, only MLR retained a statistically significant association with PI. Each 0.1-unit increase in MLR was associated with an approximately two-fold increase in the odds of parametrial involvement (adjusted OR 1.986; 95% CI 1.095–3.603; *p* = 0.024). PLR, NLR, SIR, and SIRI did not remain statistically significant after adjustment. These findings indicate that MLR may capture an inflammatory signal associated with local tumor spread, while also supporting cautious interpretation given the exploratory nature and limited sample size of the study.

## 4 Discussion

### 4.1 Interpretation of results

The current analysis shows that PI is associated with measurable changes in systemic inflammatory markers, indicating that tumor progression beyond the cervix is accompanied by a detectable host response. Biologically, PI represents a transitional phase in which the tumor spreads through direct tissue invasion and via lymphatic and vascular routes, thereby increasing its exposure to immune surveillance. This interaction between tumor tissue and the host environment may promote the activation of inflammatory pathways, as evidenced by changes in circulating blood cells (11–13). Notably, many of the evaluated indices are influenced by neutrophil activity, as neutrophils are among the earliest components of the innate immune response activated during tissue injury or tumor spread (11). This likely explains why indices that include neutrophil counts, such as NLR, consistently associate with PI. Furthermore, the significant variation observed in MLR, NLR, and SIRI across categories of parametrial involvement suggests that the systemic inflammatory response may vary with the extent of local tumor spread, although this finding remains exploratory. Exploratory ROC analysis showed only modest unadjusted discrimination, with MLR achieving the highest AUC but remaining insufficient to support clinical threshold derivation or diagnostic application. To address the limited sample size and reduce the risk of model overfitting, analyses were adjusted to use separate, clinically predefined models, each evaluating a single continuous inflammatory index in relation to depth of stromal infiltration and fornix involvement. Within this framework, only MLR retained a statistically significant association with PI after adjustment. This finding suggests that MLR may reflect a component of the systemic inflammatory response associated with local tumor spread, although it should not be interpreted as an independently validated diagnostic marker. The present analysis was not intended to develop a clinical prediction model. Accordingly, data-driven variable selection and threshold-based categorization of inflammatory indices were avoided in the revised analysis. Instead, a restricted set of anatomically relevant covariates was selected *a priori* to provide a clinically interpretable and more methodologically conservative assessment of the association. FIGO stage was not included in the adjusted models because parametrial involvement constitutes a defining component of cervical cancer stage; therefore, adjusting for stage in an analysis with parametrial involvement as the outcome would introduce circularity. Maximum tumor size was not included in the present parsimonious association-based models, which were intentionally restricted to depth of stromal infiltration and fornix involvement as predefined anatomical correlates of local spread. Although the horizontal dimension is no longer used to define the upper boundary of FIGO stage IA disease, maximum tumour size remains clinically relevant in cervical cancer; therefore, the absence of adjustment for this variable should be acknowledged as a source of potential residual confounding. Overall, the persistence of the association for MLR, together with the attenuation of the other inflammatory indices after adjustment, supports the hypothesis that systemic inflammation may accompany parametrial involvement, yet remain embedded within a broader, multifactorial process of disease progression.

### 4.2 Clinical interpretation

From a clinical perspective, the present findings should be interpreted as evidence of an association between systemic inflammatory changes and parametrial involvement rather than as evidence supporting the immediate clinical use of these indices. Complete blood counts are routinely available and inexpensive; however, the availability of these markers alone does not establish diagnostic utility. MRI, PET, and standard clinical-pathological staging procedures remain the reference approaches for assessing local tumor spread. In the present study, inflammatory indices should be interpreted exclusively as exploratory correlates of local tumor spread and not as tools for selecting patients for imaging, modifying staging procedures, or guiding treatment decisions. Inflammatory indices were intentionally retained as continuous variables in adjusted models, since no externally validated threshold is available for clinical application in this setting and the present cohort was not intended for threshold derivation or prediction-model development. Taken together, these findings suggest that systemic inflammatory indices may reflect tumor-related inflammatory burden rather than provide specific evidence of an anatomical pattern of invasion, and should therefore be interpreted only within a broader clinical and pathological context.

### 4.3 Comparison with existing literature

Compared with the existing literature, the magnitude of the differences observed in NLR and MLR is consistent with previous reports in solid tumors, where elevated values have been associated with more advanced disease and increased tumor aggressiveness. However, the exploratory standalone discrimination observed in our cohort was modest, with ROC curves indicating limited ability of individual indices to separate patients according to PI status. This finding supports a biological association with tumor spread but does not justify deriving diagnostic thresholds or proposing these markers as standalone clinical tools. Rather, their roles appear complementary, reflecting the systemic response to tumor burden rather than directly indicating specific patterns of local invasion, such as parametrial involvement. In this context, our results support the hypothesis that inflammatory indices capture a general tumor-host interaction rather than a site-specific phenomenon. The link between systemic inflammation and tumor progression observed in this study aligns with a broader oncological framework where inflammation is recognized as a key driver of tumor behavior. Similar patterns have been reported across different solid tumors, with variations in inflammatory indices connected to tumor burden, invasiveness, and prognosis (12–14). In gynecologic oncology, our group has already demonstrated similar relationships in endometrial cancer and adnexal masses, showing that systemic inflammatory indices reflect underlying tumor characteristics and progression dynamics (15–17). These findings support the idea that the connection between inflammation and tumor spread may be a common biological mechanism across different tumor types rather than a disease-specific phenomenon (18–20).

### 4.4 Strengths and limitations

The study highlights several strengths, including a clearly defined analytical framework centered on a specific clinical question and the use of a well-characterized cohort with consistent data collection. The methodological approach, which combines comparative analyses, adjusted association models, and exploratory assessment of unadjusted discrimination, enables a structured evaluation of the relationship between inflammatory indices and PI without proposing a diagnostic prediction tool. Although parametrial involvement was assessed using both histopathological and radiological methods, only a very small proportion of patients (2.5%) were evaluated by imaging, thus minimizing potential heterogeneity. However, some limitations should be noted. The study population is limited to a specific geographic area, which may expose patients to shared environmental factors and dietary habits that could influence systemic inflammatory profiles and limit external generalizability. A significant imbalance in treatment distribution between groups was observed. Although treatment distribution differed between groups, this variable was not considered a confounder in the present analysis, as all inflammatory indices were measured prior to treatment initiation. Therefore, treatment modality cannot have influenced the exposure variables, although it may reflect underlying disease severity. The relatively small sample size represents an important limitation and may affect the precision and stability of adjusted effect estimates. To address this issue, inflammatory indices were retained as continuous variables and evaluated in separate adjusted models, avoiding both information loss related to dichotomization and the simultaneous inclusion of multiple potentially correlated inflammatory measures. Moreover, adjustment was deliberately restricted to depth of stromal infiltration and fornix involvement, which were selected *a priori* as clinically relevant indicators of local anatomical spread. Moreover, ROC analyses were retained only for descriptive summary of unadjusted discrimination. No cut-off values or threshold-based diagnostic accuracy estimates were included in the revised analysis, because any such estimates derived and evaluated within the same limited cohort would be prone to optimism and would require independent validation. Finally, because the study is retrospective, the findings should be seen as associative rather than causal, and further validation in independent cohorts is necessary to confirm their reliability.

## 5 Conclusion

In patients with cervical cancer, parametrial involvement is associated with measurable changes in routinely available systemic inflammatory indices, including PLR, MLR, NLR, and SIRI. In revised adjusted analyses based on separate clinically predefined models using continuous variables, only MLR retained a statistically significant association with PI after adjustment for depth of stromal infiltration and fornix involvement. Nevertheless, the modest standalone discriminative performance and exploratory nature of the study indicate that inflammatory indices should be regarded as complementary markers of tumor-related inflammatory response rather than independent diagnostic tools. Larger prospective studies are required to validate the association observed for MLR and to determine whether inflammatory indices may contribute to multimodal assessment strategies.

## Data Availability

The datasets presented in this study can be found in online repositories. The names of the repository/repositories and accession number(s) can be found in the article/Supplementary material.
