# Supplementary material for: Parametrial involvement in cervical cancer is associated with systemic inflammatory indices: a retrospective observational study
Source: Front Med (Lausanne). 2026 Jun 25;13:1842260. doi: 10.3389/fmed.2026.1842260 (PMC13346039; doi:10.3389/fmed.2026.1842260)
Supplement: Supplementary file 1 [file Table_1.docx]

Supplementary Material

# Supplementary Data

A. Diagnostic performance of NLR in predicting parametrial involvement

| Measure | Estim. | Low.lim.95.. | Up.lim.95.. |
| --- | --- | --- | --- |
| Sensitivity | 0.512 | 0.351 | 0.671 |
| Specificity | 0.868 | 0.719 | 0.956 |
| Pos.Pred.Val. | 0.808 | 0.606 | 0.934 |
| Neg.Pred.Val. | 0.623 | 0.479 | 0.752 |
| LR+ | 3.893 | 1.631 | 9.289 |
| LR- | 0.562 | 0.401 | 0.787 |
| Odds ratio | 6.930 | 2.256 | 21.290 |
| Youden index | 0.381 | 0.194 | 0.568 |
| Accuracy | 0.684 | 0.569 | 0.784 |
| Error rate | 0.316 | 0.216 | 0.431 |

B. Diagnostic performance of MLR in predicting parametrial involvement

| Measure | Estim. | Low.lim.95.. | Up.lim.95.. |
| --- | --- | --- | --- |
| Sensitivity | 0.902 | 0.769 | 0.973 |
| Specificity | 0.447 | 0.286 | 0.617 |
| Pos.Pred.Val. | 0.638 | 0.501 | 0.760 |
| Neg.Pred.Val. | 0.810 | 0.581 | 0.946 |
| LR+ | 1.633 | 1.206 | 2.211 |
| LR- | 0.218 | 0.081 | 0.590 |
| Odds ratio | 7.488 | 2.225 | 25.204 |
| Youden index | 0.350 | 0.167 | 0.532 |
| Accuracy | 0.684 | 0.569 | 0.784 |
| Error rate | 0.316 | 0.216 | 0.431 |

C. Diagnostic performance of PLR in predicting parametrial involvement

| Measure | Estim. | Low.lim.95.. | Up.lim.95.. |
| --- | --- | --- | --- |
| Sensitivity | 0.683 | 0.519 | 0.819 |
| Specificity | 0.605 | 0.434 | 0.760 |
| Pos.Pred.Val. | 0.651 | 0.491 | 0.790 |
| Neg.Pred.Val. | 0.639 | 0.462 | 0.792 |
| LR+ | 1.730 | 1.108 | 2.701 |
| LR- | 0.524 | 0.312 | 0.879 |
| Odds ratio | 3.303 | 1.309 | 8.329 |
| Youden index | 0.288 | 0.077 | 0.499 |
| Accuracy | 0.646 | 0.530 | 0.750 |
| Error rate | 0.354 | 0.250 | 0.470 |

D. Diagnostic performance of SIR in predicting parametrial involvement

| Measure | Estim. | Low.lim.95.. | Up.lim.95.. |
| --- | --- | --- | --- |
| Sensitivity | 0.951 | 0.835 | 0.994 |
| Specificity | 0.289 | 0.154 | 0.459 |
| Pos.Pred.Val. | 0.591 | 0.463 | 0.710 |
| Neg.Pred.Val. | 0.846 | 0.546 | 0.981 |
| LR+ | 1.339 | 1.080 | 1.659 |
| LR- | 0.169 | 0.040 | 0.712 |
| Odds ratio | 7.944 | 1.629 | 38.745 |
| Youden index | 0.241 | 0.082 | 0.399 |
| Accuracy | 0.633 | 0.517 | 0.739 |
| Error rate | 0.367 | 0.261 | 0.483 |

E. Diagnostic performance of SIRI in predicting parametrial involvement

| Measure | Estim. | Low.lim.95.. | Up.lim.95.. |
| --- | --- | --- | --- |
| Sensitivity | 0.732 | 0.571 | 0.858 |
| Specificity | 0.553 | 0.383 | 0.714 |
| Pos.Pred.Val. | 0.638 | 0.485 | 0.773 |
| Neg.Pred.Val. | 0.656 | 0.468 | 0.814 |
| LR+ | 1.636 | 1.097 | 2.438 |
| LR- | 0.485 | 0.272 | 0.868 |
| Odds ratio | 3.369 | 1.314 | 8.636 |
| Youden index | 0.284 | 0.076 | 0.493 |
| Accuracy | 0.646 | 0.530 | 0.750 |
| Error rate | 0.354 | 0.250 | 0.470 |

**
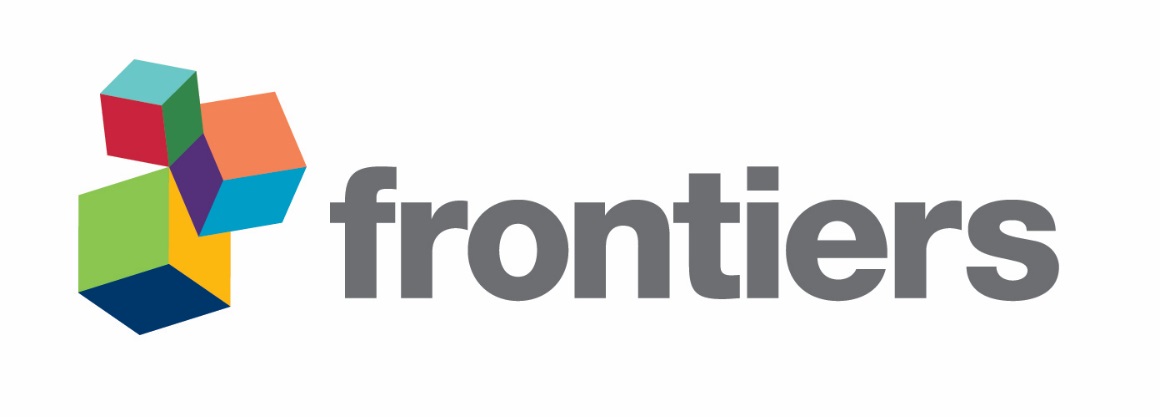
**
